# Supplementary material for: The Acinetobacter baumannii disinfectant resistance protein, AmvA, is a spermidine and spermine efflux pump
Source: Commun Biol. 2021 Sep 22;4:1114. doi: 10.1038/s42003-021-02629-6 (PMC8458285; doi:10.1038/s42003-021-02629-6)
Supplement: Supplementary file 2 — Supplementary information [file 42003_2021_2629_MOESM2_ESM.pdf]

Supplementary Information

Supplementary Figures
Supplementary Figure 1 Divergent regulatory and phenotypic effects of polyamines
Supplementary Figure 2 Mutation of *amvA*, *amvR* or *adeB* does not alter tolerance to short-chain polyamines.
Supplementary Figure 3 Effect of polyamines and AmvR on expression of an *amvA*$_{prom}$-GFP reporter.
Supplementary Figure 4 Purification and binding studies of AmvR

Supplementary Tables
Supplementary Table 1 Sequencing statistics
Supplementary Table 2 Polyamine-regulated genes of interest
Supplementary Table 3 Summary of isothermal analysis with fitted ΔC_p
Supplementary Table 4 Summary of isothermal analysis with ΔC_p set to zero
Supplementary Table 5 Bacterial strains used in this study
Supplementary Table 6 Plasmids used in this study
Supplementary Table 7 Oligonucleotides used in this study

Supplementary References

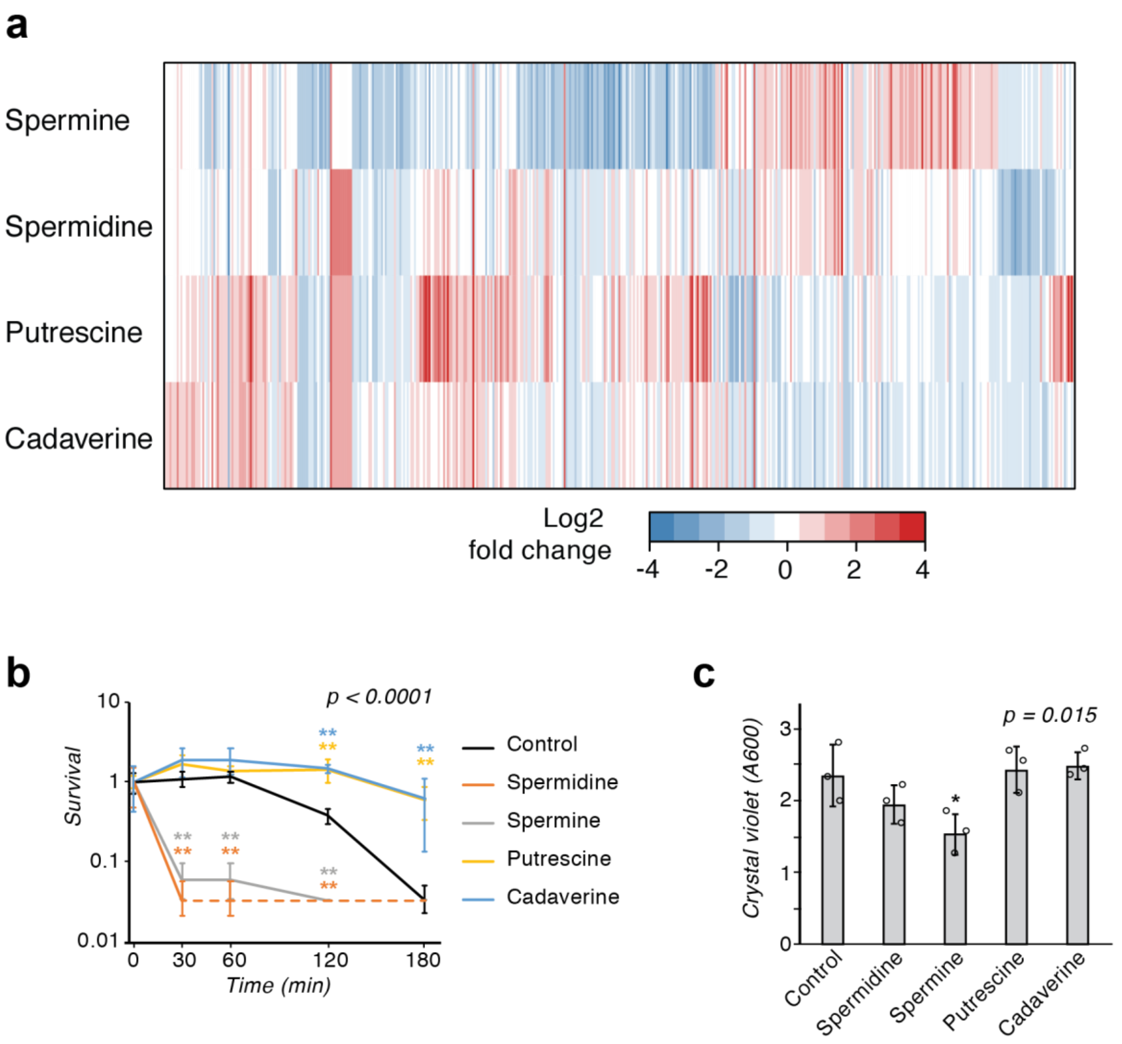

Supplementary Figure 1. Divergent regulatory and phenotypic effects of polyamines (A) Heatmap of expression changes following polyamine shock, showing the direction of expression change, and concordance between different polyamines. (B) Serum survival curves following treatment of 10^6 cells with 66% normal human serum supplemented with polyamines at 1/8 MIC (n = 3 biological replicates). The chart shows geometric mean and standard deviation. Statistical significance was determined by two-factor repeated measures ANOVA on $\log_{10}$-transformed survival data (overall p-value < 0.0001) followed by a one-factor ANOVA with Dunnett's post-hoc test at individual time points to compare all samples to the control. **p < 0.01. (C) Biofilm formation in the presence of polyamines (n = 3 biological replicates). Static 2.5ml cultures with or without polyamine supplementation were incubated for 40 hours at 37C, and the attached biomass quantified by crystal violet staining. Shown are the mean and standard deviation for each treatment. *p < 0.05, one-way ANOVA with Dunnett's post-hoc test (overall p-value = 0.015).

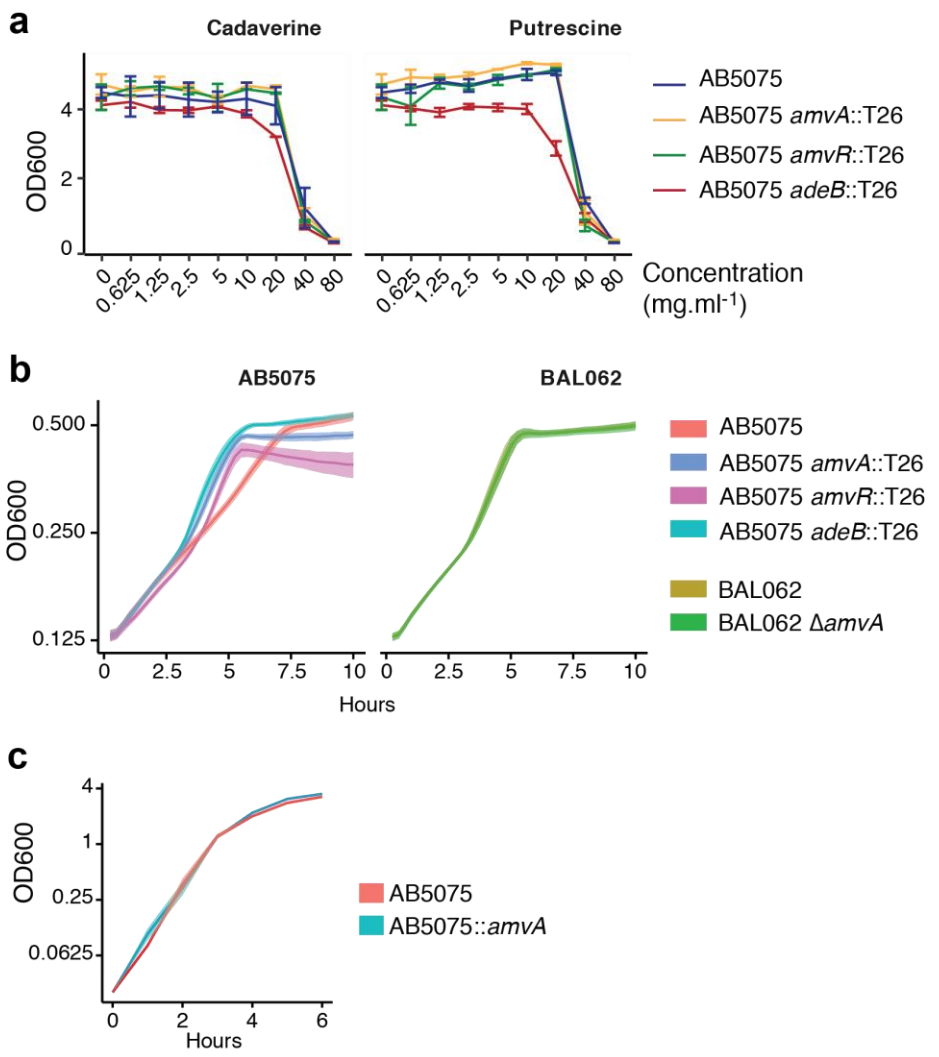

Supplementary Figure 2. Mutation of *amvA*, *amvR* or *adeB* does not alter tolerance to short-chain polyamines. (A) Cadaverine and putrescine MIC determination with *A. baumannii* AB5075 and mutant strains, mean ± standard deviation for two biological and two technical replicates. (B) Growth curve of *A. baumannii* AB5075 and BAL062 and mutants in 96-well plate format with shaking every 15 min. Results shown are mean ± standard deviation from three biological replicates, each comprised of five technical replicates (C) Growth curve of *A. baumannii* AB5075 and tn::*amvA* mutant in aerobic conditions. Results are mean ± standard deviation from three biological replicates.

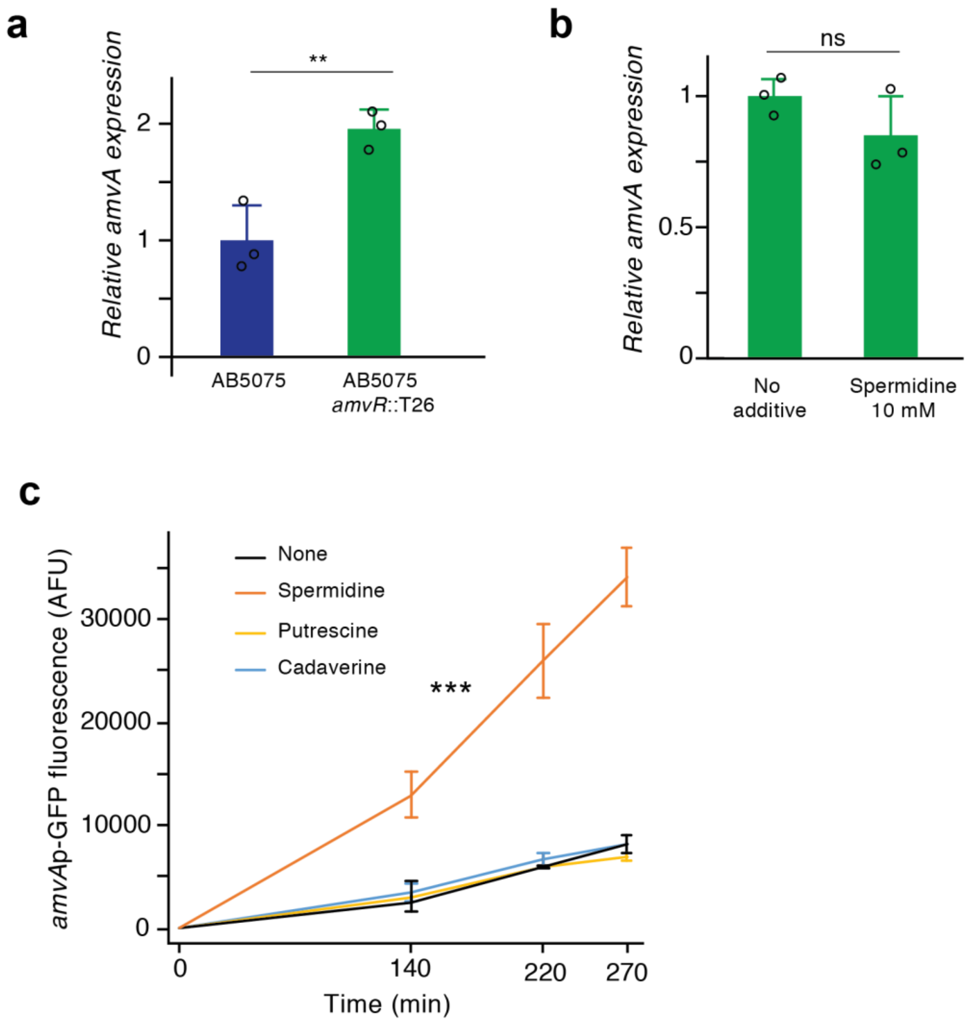

Supplementary Figure 3. Effect of polyamines and AmvR on expression of an *amvA*$_{\text{prom}}$-GFP reporter. (A) Fluorescence of GFP in *A. baumannii* AB5075 or *A. baumannii* AB5075-tn-*amvR* carrying the *amvA* expression reporter vector pFLS45. Reporter fluorescence was measured in cultures grown aerobically to OD = 2. Results shown are the mean ± standard deviation from three biological replicates, ** $p < 0.01$, one-way ANOVA. (B) Induction of *amvA*prom-GFP expression by SPD does not occur in AB5075 tn-*amvR*. Results are mean ± standard deviation for three biological replicates. (C) Induction of *amvA* expression in *A. baumannii* ATCC17978 pFLS43 by 10 mM cadaverine, putrescine or spermidine. Note that cells did not grow when 10 mM spermine was added. Results shown are mean ± standard deviation from three biological replicates, each comprised of five technical replicates. Each polyamine-treated data series was compared to the untreated control by mixed-repeated measures ANOVA, *** $p < 0.0001$.

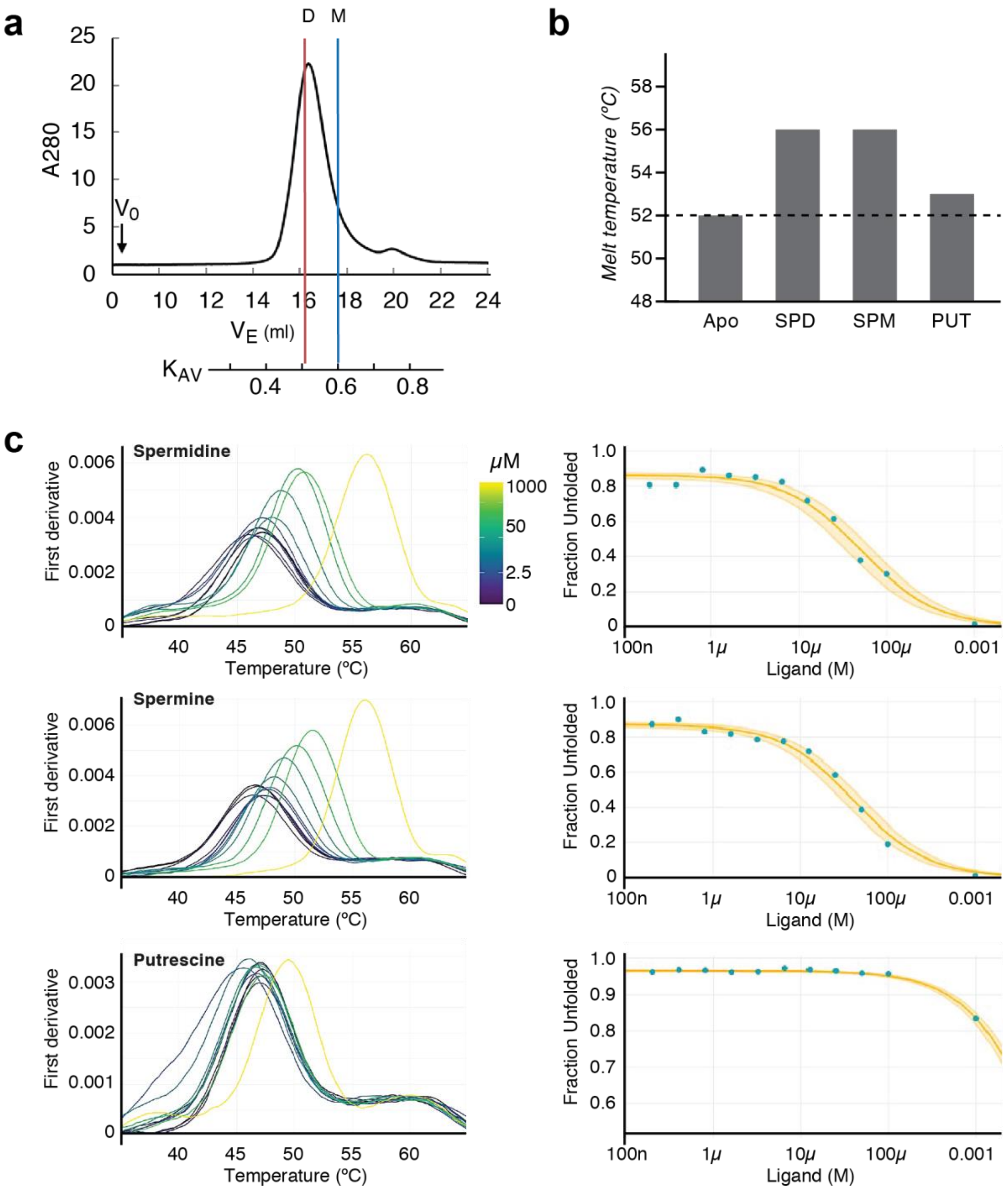

Supplementary Figure 4. Purification and DSF analysis of AmvR. (A) Analytical size exclusion chromatography trace for AmvR on Superdex 200. V_T = 24 ml, V_0 = 8.34 ml. Predicted elution positions are indicated for the theoretical dimer (D) and monomer (M) forms. (B) Shifts in melting temperature of purified AmvR in the presence of polyamines. Tm increases of 4 °C were seen in the presence of spermine and spermidine, while putrescine caused a 1°C shift. Experiments were performed in triplicate and gave identical Tm values. (C) Derivative (left) and fitted (right) nanoDSF isothermal analysis measurements for AmvR in the presence of spermine, spermidine and putrescine. This experiment is a replicate of that in Figure 4B.

Supplementary Table 1 RNA sequence-reads and overall alignment rate in *ABUW-5075*

Polyamines	Average sequence reads	Overall alignment rate
Spermidine tri-hydrochloride	11,090,209 ± 210,756	98.77%
Spermine tetra-hydrochloride	10,414,484 ± 541,477	98.82%
Putrescine di-hydrochloride	11,194,537 ± 1,155,240	99.19%
Cadaverine di-hydrochloride	10,833,984 ± 487,424	98.96%

Supplementary Table 2 Polyamine-regulated genes of interest (xlsx). Abbreviations: PUT = putrescine, CAD = cadaverine, SPD = spermidine, SPM = spermine. Shown are locus tags, functional categories, specific regulation by polyamines, and any related enriched GO terms.

Locus tag(s)	Function	Induced by	Repressed by	Significant GO term enrichments
Metabolism				
Amino acid catabolism				
ABUW_0069-71	Phe degradation/transport	PUT	SPM	GO:0006559 L-phenylalanine catabolic process (PUT)
ABUW_0077-81	Histidine utilisation	PUT (ABUW_0077-81); SPD (ABUW_0077-8)		GO:0006548 Histidine catabolic process (PUT)
ABUW_2451-2456	L-leucine degradation	PUT (ABUW_2451-6); SPD (ABUW_2452-3); CAD (ABUW_2454-6)	SPM	GO:0006552 leucine catabolic process (PUT, CAD, SPD, SPM)
Lipid metabolism				
ABUW_1572-4	acyl-CoA dehydrogenase/ligase/ short-chain dehydrogenase	SPD; PUT; CAD		
ABUW_2095-9	*ato* fatty acid utilisation operon	PUT (ABUW_2096-9); CAD (ABUW_2096-7)	SPM	
Aerobic respiration				
ABUW_1792-1795	cytochrome D operon		PUT	
ABUW_3733-4	ATP synthase F1		PUT	
Other metabolic pathways				
ABUW_0201, 0203-4	GABA degradation	PUT		

Locus tag(s)	Function	Induced by	Repressed by	Significant GO term enrichments
ABUW_1831-9	protocatechuate utilisation	PUT (ABUW_1831-9); SPD (ABUW_1831-4)		
ABUW_1840-1	quinate degradation	PUT		
Virulence, resistance and HGT				
Iron acquisition				
ABUW_0159	TonB siderophore uptake	SPD; SPM		GO:0006826 iron ion transport (SPM)
ABUW_1170, 1175-77	Acinetobactin transport	SPM		GO:0044718 siderophore transmembrane transpot (SPM)
ABUW_1355	hemin uptake (hemP)	SPD; SPM; PUT		
ABUW_2184-9	aerobactin	SPM (ABUW_2184-9); SPD (ABUW_2185-9); PUT (ABUW_2188-9)		GO:0019270 aerobactin biosynthetic process (SPD, SPM)
ABUW_2324	heme oxygenase-like protein		SPM	
ABUW_3125	bacterioferritin		SPD; SPM	
ABUW_3632-3	ferrous iron transport feoAB	SPD (ABUW_3632-3); PUT (ABUW_3633)		
Motility, adhesion and defense				
ABUW_0291-292, 304, 3031-2	Type IV pilus		PUT (ABUW_0291-2, 304, 3031-2); CAD (ABUW_0291-2)	GO:0043107 Type IV pilus-dependent motiliy (PUT)
ABUW_0678, 80	Type IV pilus (response regulator)		PUT	
ABUW_1488-91	Csu pilus		SPM (ABUW_1491); PUT (ABUW_1489); CAD (ABUW_1488-9)	GO:0009297 pilus assembly (CAD)
ABUW_2572-5	Type VI secretion		SPM (ABUW_2572-5); CAD (ABUW_2576-7)	
ABUW_1088-1090, 1096	Type I-F CRISPR		SPM	

Locus tag(s)	Function	Induced by	Repressed by	Significant GO term enrichments
Resistance functions				
ABUW_1673	aceI	SPD; PUT; CAD		
ABUW_1678	amvR (regulator)	SPD; SPM; PUT; CAD		
ABUW_1679	smvA	SPD; SPM		
ABUW_1974-6	adeABC efflux pump	SPD; SPM; PUT; CAD		
ABUW_1352	Peroxide stress response		SPD; SPM; PUT; CAD	GO:0070301 cellular response to hydrogen peroxide
ABUW_2436	katE	PUT; CAD		GO:0070301 cellular response to hydrogen peroxide (CAD)
ABUW_3227-8	pcoAB copper resistance protein	SPM		
ABUW_3320-2, ABUW_3325	copAB copper resistance, copper-translocating P-type ATPase	PUT; CAD		GO:0035434 copper ion transmembrane transport
Unknown				
ABUW_0233		SPD; SPM; PUT; CAD		
Prophage genes				
ABUW_0741-91	Prophage region 2		SPD (ABUW_0741, 52, 0756, 0758, 0762-4, 0774, 0780, 0791)	
ABUW_1255-1286	Prophage region 3	PUT (ABUW_1255-60, 1266-7, 1286)	SPM (ABUW_1261-63)	
ABUW_1297-1311	Prophage region 4		SPD (ABUW_1297); SPM (ABUW_1298, 1301, 1304, 1309, 1311)	
ABUW_1394-1439	Prophage region 5		SPD (ABUW_1394, 1414-5, 1421, 1425, 1438-9)	
ABUW_2655-2673	Prophage region 6	SPD (ABUW_2669); SPM (ABUW_2669)	SPM (ABUW_2655, 2666, 2673); PUT (ABUW_2665);	

**Locus tag(s)**	**Function**	**Induced by**	**Repressed by**	**Significant GO term enrichments**
			CAD (ABUW_2666)	

Supplementary Table 3 Summary of isothermal analysis with fitted ΔC_p

	Replicate 1			Replicate 2		
AmvR (15 µM)	Heating rate	2 °C/min		Heating rate	1.5 °C/min	
	ΔC_p	T_{sel}	$K_{d,\ Tsel}$ (Ratio)	ΔC_p	T_{sel}	$K_{d,\ Tsel}$ (Ratio)
Spermidine	Fitted: 8.34 kcal/mol	49 °C	9.5 µM ± 13%	Fitted: 9.61 kcal/mol	49 °C	6.8 µM ± 21%
Spermine	Fitted: 8.34 kcal/mol	49 °C	5.4 µM ± 13%	Fitted: 10.73 kcal/mol	49 °C	5.2 µM ± 17%
Putrescine	Fitted: 7.57 kcal/mol	51 °C	260 µM ± 6%	Fitted: 7.18 kcal/mol	51 °C	220 µM ± 8%

T_{sel} is the temperature with the lowest fitting error for K_d

Supplementary Table 4 Summary of isothermal analysis with ΔC_p set to zero

	Replicate 1			Replicate 2		
AmvR (15 µM)	Heating rate	2 °C/min		Heating rate	1.5 °C/min	
	ΔC_p	T_{sel}	$K_{d,\ Tsel}$ (Ratio)	ΔC_p	T_{sel}	$K_{d,\ Tsel}$ (Ratio)
Spermidine	Default: 0 kcal/mol	49 °C	10 µM ± 17%	Default: 0 kcal/mol	49 °C	8 µM ± 20%
Spermine	Default: 0 kcal/mol	49 °C	5.8 µM ± 15%	Default: 0 kcal/mol	49 °C	6.1 µM ± 18%
Putrescine	Default: 0 kcal/mol	51 °C	310 µM ± 6%	Default: 0 kcal/mol	51 °C	330 µM ± 7%

T_{sel} is the temperature with the lowest fitting error for K_d

Supplementary Table 5 Bacterial strains used in this study

Strain	Description/genotype	Source
Acinetobacter baumannii AB5075-UW	Drug-resistant isolate, International clonal lineage 1	(1)
Acinetobacter baumannii ATCC17978	Drug-susceptible isolate	ATCC
Escherichia coli DH5α	Cloning strain, F^- ϕ80*lac*ZΔM15 Δ(*lac*ZYA-*arg*F)U169 *rec*A1 *end*A1 *hsd*R17(r_K^-, m_K^+) *pho*A *sup*E44 λ^- *thi*-1 *gyr*A96 *rel*A1	Invitrogen
Escherichia coli BL21(DE3)	Protein expression strain, F^- *omp*T *hsd*S_B (r_B^-, m_B^-) *gal* *dcm* (DE3)	New England Biolabs
Acinetobacter baumannii AB5075-UW *amvA*::T26	AB5075-UW ABUW_1679::420(+)T26 (*amvA*), item AB04444	(2)
Acinetobacter baumannii AB5075-UW *amvR*::T26	AB5075_UW ABUW-1678::263(+)T26 (*amvR*), item AB04443	(2)
Acinetobacter baumannii AB5075-UW *adeB*::T26	AB5075-UW ABUW_1975::1030(+)T26 (*adeB*), item AB05228	(2)
Acinetobacter baumannii BAL062	Isolate of international clonal lineage 2	(3)
Acinetobacter baumannii BAL062 Δ*amvA*	Deletion mutant of *amvA*, constructed by linear fragment transformation with construct generated with primers AP2064 and FS175-179	This study

Supplementary Table 6 Plasmids used in this study

Name	Description	Source
pVRL1	*E. coli-A. baumannii* shuttle vector, Gm^R	(4)
pVRL1-Z	*E. coli-A. baumannii* shuttle vector, Zeo^R	(4)
pFLS43	GFP with *amvA* promoter (100bp) in pVRL1	This study
pFLS45	GFP with *amvA* promoter (100bp) in pVRL1-Z	This study
$pTTQ18_{RGSH6}$	IPTG-inducible expression vector, C-terminal 6xHis tag	(5)
$pTTQ18_{RGSH6}$-*amvA*	*amvA* gene (previously named *aedF*) from ATCC17978 cloned into pTTQ18	(6)
pAmvR	AmvR gene from ATCC17978 cloned into $pTTQ18_{RGSH6}$	This study
pCR2.1	TOPO cloning vector, Amp^R	Invitrogen
pDiGc	Fluorescence reporter vector with GFP and dsRED; GFP source	(7)

Supplementary Table 7 Oligonucleotides used in this study

**Name**	**Sequence (5'-3' direction)**	**Description**
AmvR-F	ttcacacaggaaacagcgatggcctatcttaatcgcgat	AmvR gene Fwd
AmvR-R	cggccacctctgcagccggattggaagtacaggttctctaataattctaggc g	AmvR gene Rev
AP2064	gtgtggatccatgattccaaaaggaatgaa	*amvA* linear fragment mutagenesis
FS09	agaggagctcgatcccggagttcatgc	pDiGc marker fragment Fwd
FS10	gtgtctcgagagagtttgtagaaacgcaaaaagg	pDiGc marker fragment Rev
FS65	tgcgagatacccagatcacatgaaacagcatgactttttc	GFP overlap primer Fwd
FS66	gtcatgctgtttcatgtgatctgggtatctcgca	GFP overlap primer Rev
FS76	gagacatatggtttgttacacctgactg	*amvA-amvR* intergenic Fwd
FS77	gagacatatgaacgctcttcgatatcca	*amvA-amvR* intergenic Rev
FS175	gtgtggatccatgattccaaaaggaatgaa	*amvA* linear fragment mutagenesis
FS176	atcgaagagcgttatgccggaattgccagctggggc	*amvA* linear fragment mutagenesis
FS177	tactttcttcggaaattcagaagaactcgtcaagaaggc	*amvA* linear fragment mutagenesis
FS178	cgagttcttctgaatttccgaagaaagtaaattaatatttttc	*amvA* linear fragment mutagenesis
FS179	ctaaattggagtaataactggctcac	*amvA* linear fragment mutagenesis
qPCR_amvA_F	ccatgattgctttggttggc	qPCR primer
qPCR_amvA_R	acctgctaatggaccacct	qPCR primer
qPCR_gadph_F	caacactggtaaatggcgtg	qPCR primer
qPCR_gadph_R	acaacgtttttcatttcggc	qPCR primer

Supplementary references

1. Jacobs AC, Thompson MG, Black CC, Kessler JL, Clark LP, McQueary CN, Gancz HY, Corey BW, Moon JK, Si Y, Owen MT, Hallock JD, Kwak YI, Summers A, Li CZ, Rasko DA, Penwell WF, Honnold CL, Wise MC, Waterman PE, Lesho EP, Stewart RL, Actis LA, Palys TJ, Craft DW, Zurawski D V. 2014. AB5075, a Highly Virulent Isolate of Acinetobacter baumannii, as a Model Strain for the Evaluation of Pathogenesis and Antimicrobial Treatments. MBio 5:e01076-14.
2. Gallagher LA, Ramage E, Weiss EJ, Radey M, Hayden HS, Held KG, Huse HK, Zurawski D V., Brittnacher MJ, Manoil C. 2015. Resources for genetic and genomic analysis of emerging pathogen Acinetobacter baumannii. J Bacteriol 197:2027–2035.
3. Hassan KA, Cain AK, Huang T, Liu Q, Elbourne LDH, Boinett CJ, Brzoska AJ, Li L, Ostrowski M, Nhu NTK, Nhu TDH, Baker S, Parkhill J, Paulsen IT. 2016. Fluorescence-based flow sorting in parallel with transposon insertion site sequencing identifies multidrug efflux systems in acinetobacter baumannii. MBio 7:3–8.
4. Lucidi M, Runci F, Rampioni G, Frangipani E, Leoni L, Visca P. 2018. New shuttle vectors for gene cloning and expression in multidrug-resistant Acinetobacter species. Antimicrob Agents Chemother 62:1–19.
5. Stark MJ. 1987. Multicopy expression vectors carrying the lac repressor gene for regulated high-level expression of genes in Escherichia coli. Gene 51:255–267.
6. Hassan KA, Brzoska AJ, Wilson NL, Eijkelkamp BA, Brown MH, Paulsen IT. 2011. Roles of DHA2 family transporters in drug resistance and iron homeostasis in Acinetobacter spp. J Mol Microbiol Biotechnol 20:116–124.
7. Helaine S, Thompson JA, Watson KG, Liu M, Boyle C, Holden DW. 2010. Dynamics of intracellular bacterial replication at the single cell level. Proc Natl Acad Sci U S A 107:3746–3751.
