# Supplementary material for: The Acinetobacter baumannii disinfectant resistance protein, AmvA, is a spermidine and spermine efflux pump
Source: Commun Biol. 2021 Sep 22;4:1114. doi: 10.1038/s42003-021-02629-6 (PMC8458285; doi:10.1038/s42003-021-02629-6)
Supplement: Supplementary file 3 — Description of Supplementary Files [file 42003_2021_2629_MOESM3_ESM.pdf]

**Description of Additional Supplementary Files**

**File name:** Supplementary Data 1

**Description:** Polyamine-induced gene expression changes in A. baumannii AB5075.

**File name:** Supplementary Data 2

**Description:** A. baumannii pangenome gene presence-absence table.

**File name:** Supplementary Data 3

**Description:** Source data for figures.
